# Deinfibulation Contextualized: Delicacies of Shared Decision-Making in the Clinic

**DOI:** 10.1007/s10508-020-01676-0

**Published:** 2020-03-13

**Authors:** Sara Johnsdotter, Birgitta Essén

**Affiliations:** 1 Centre for Sexology and Sexuality Studies, Faculty of Health and Society, Malmö University, 205 06 Malmö, Sweden; 2 Department of Women’s and Children’s Health, International Maternal and Child Health, Uppsala University, Uppsala, Sweden

The proposed model for clinical conversations about infibulation that Brady, Connor, Chaisson, Mohamed, and Robinson (2019) present in their Target Article encompasses a refreshingly nonjudgmental approach to dealing with deinfibulation as a clinical phenomenon and as an object of study. The merits of their approach are manifold: They recommend a truly sensitive and respectful attitude toward women with female genital cutting (FGC), and they highlight the urgency of seeing the wider context that may impact women’s choices in order to promote shared decision making in clinical encounters. Their lack of prejudice even makes them suggest that the diversity of genital appearance should be framed as healthy and beautiful. This is a crucial message in a time when global anti-FGM activism, ubiquitous in Western host countries, sends messages telling migrated girls and women with FGC that they are “mutilated,” disfigured, and not fully feminine (for critical discussions of this state of affairs, see, e.g., Ahmadu, 2007; Catania, Abdulcadir, Puppo, Verde, Abdulcadir, & Abdulcadir, 2007; Johnsdotter, 2020; Johnsdotter & Essén, 2015; Johnson-Agbakwu & Warren, 2017; Malmström, 2013; Villani, 2009).

Our overall point of view is thus very positive: We endorse the effort to create a framework that handles the question of deinfibulation with such nuance and respect for the affected girls and women. Our main concerns with Brady et al.’s (2019) Target Article regard how medical consequences of FGC are framed, as well as how their model and discussion disregard the importance of cultural change after migration and the effects of anti-FGM policies in Western countries.

## The Framing of Medical Consequences of Female Genital Cutting

For decades, the World Health Organization has highlighted the possible medical consequences of FGC in their fervor to spread information on the harm of FGC (e.g., WHO, 1995, 2010, 2014). Some scholars have raised the issue of how weak the actual research evidence is in relation to the representation of the medical sequalae (Essén, Sjöberg, Gudmundsson, Östergren, & Lindqvist, 2005; Hodžić, 2013; Morison et al., 2001; Obermeyer, 1999, 2003, 2005; PPAN, 2012). Even in articles that clearly frame FGC as a very harmful practice, the figures establish the fact that far from all women with FGC, or even with type III infibulation, suffer from chronic pain or other symptoms (e.g., the survey by Berg, Underland, Odgaard-Jensen, Fretheim, & Vist, 2014). Furthermore, surveys and meta-studies of medical consequences are based on studies that are limited in scope and often of poor quality (Berg et al., 2014), which has implications for the quality of the meta-analyses presented. A few lines from a table in an article about care practices are illustrative: “Deinfibulation is recommended [by WHO] for preventing and treating obstetric complications in women living with type 3 FGM [strong recommendation; very low-quality evidence]” (von Rège & Campion, 2017, p. 25).

Yet, the expectation that medical consequences are inevitable is often the unquestioned starting point. In the Target Article by Brady et al. (2019), they refer to the WHO recommendation of deinfibulation, said to “prevent and treat gynecological complications” and maximize health, and they state that the procedure ought to be done to “alleviate the most pronounced physical effects of type III FGC, a cultural practice.” Even though Brady et al. stress the importance of open-mindedness and rapport in the clinical encounter, it is clear that they see deinfibulation as the desired outcome of the shared decision making between caregiver and patient. We would like to argue that a genuinely culturally sensitive—and professional—approach would involve a more pronounced openness toward the possibility that girls and women can live well despite the fact that they are infibulated.

There is a risk associated with ostensibly downplaying the medical consequences of FGC: One might stand out as a cynical person who does not sympathize with girls and women who have suffered bodily harm. But, perhaps needless to say, nuanced views and expectations among caregivers are crucial for the optimal outcome of a clinical encounter. Expecting and searching for complications and suffering where there are none to address may lead to unwanted clinical outcomes and encounters that are less than optimal. In addition, too strong a focus on FGC as the presumed cause of symptoms, when this is not the case, may lead to the healthcare provider’s failing to identify the real origin of a woman’s complaints.

Deinfibulation is medically essential in some clinical situations. For example, it is the preferred option when there is a need to diagnose or treat conditions that require an ocular inspection or surgery of the uterus, cervix, or vagina. A real dilemma for the caregiver might emerge if the woman and/or her next of kin refuse deinfibulation when it is essential for medical reasons: at cervical cancer screenings or assessments of a genital infection, to make a fertility evaluation possible, to facilitate monitoring the fetus during delivery with a scalp electrode, and other vaginal treatments such as labor induction, miscarriage, and induced abortion.

Moreover, if a girl or woman suffers from her infibulation in any way, deinfibulation is the obvious preferred treatment. But the clinical encounter ought to start with an unbiased clinical assessment of the individual case, in which the possibility that the patient does not need deinfibulation (at this point of her life) should be on the table. Consequently, before the conceptual model suggested by Brady et al. (2019) is set in motion, caregivers should be encouraged to use their professional discretion in order to establish whether the patient before them is one that suffers from the infibulation or not. A recent study among Somalis in Norway showed that even though attitudes to FGC in general are negative in this group, already infibulated women may prefer to preserve their state of being infibulated until a later point in life, as long as they are doing fine: “[W]hereas many informants claimed that they would accept premarital defibulation in cases of severe health problems, they considered it socially unacceptable and thus not done except in rare cases” (Johansen, 2019, p. 15). A medically based assessment needs to be performed before encouraging a patient to go through deinfibulation. If the girl or woman has no health issues related to the infibulation, it might be better to lay the issue to rest and be content with offering brief information about the option of deinfibulation forthwith or at a later stage.

If this is not the starting point, there is a risk that paternalistic attitudes—warned against by Brady et al. (2019)—will impact the encounter. If the conceptual model presented in the Target Article is launched with the ultimate motive of overcoming any possible reluctance to agree to deinfibulation, then a somewhat deceitful or manipulative attitude will be present during the encounter, and the patient will be implicitly reduced to a person who does not understand her own interests. Thus, making deinfibulation the desired outcome of every clinical conversation about infibulation is not in line with an authentic sensitive approach.

## Age as a Missing Aspect of the Framework

The proposed model’s battery of questions for conversations about infibulation is nonjudgmental and sensitive, but it lacks focus on the age of the patient. The clinical situation unfolds very differently depending on whether the patient is a girl child accompanied by her mother or other custodian, a young teenager, an adult woman who is ready for marriage, a pregnant woman prior to, or during, delivery, or an older woman. Consent, patient autonomy and other people’s impact on decision making vary greatly according to age and life situation. For example, if a child has medical issues due to infibulation, parental consent is needed for deinfibulation to take place. Conversations with adult women will unfold completely differently depending on how long they have lived in the host country. If the model suggested by Brady et al. (2019) could be adapted to address the variety of patients that present with infibulation, it would make their approach even more useful.

## The Issue of Reinfibulation and Male Sexual Pleasure

Brady et al. (2019) bring up the risk that healthcare providers “mistakenly believe that reinfibulation is desired” and, citing Johansen (2017), assert that “both women and men may wish to retain a small vaginal opening because they consider it to be a prerequisite for male sexual pleasure.” These themes, and their interconnectedness, would have benefitted from some elaboration in the Target Article by Brady et al., since such notions among caregivers about the patient group they encounter may indeed affect the care given.

Reinfibulation is not customary in Somalia—in contrast to the Sudan, where women ideally are expected to be reinfibulated after delivery (Gruenbaum, 2006; Johansen, 2006). Norwegian anthropologist Johansen (2006) reported from her study among Norwegian Somalis: “Reinfibulation was virtually unheard of among my Somali informants, and most sighed and shuddered at the thought of what they considered a cruel and primitive practice they knew to be widespread in Sudan” (p. 528). Somali women are expected to remain deinfibulated after marriage and childbirth (Johansen, 2006; Johnsdotter, 2002). Yet, caregivers in Western host countries might expect Somali women to want reinfibulation because they confuse the Sudanese practice of reinfibulation with some Somali women’s fears of being “wide open” after delivery. Brady et al. (2019) also noted that reports from caregivers about women wanting reinfibulation after delivery might be based on misunderstandings. Johansen (2006) discussed how caregivers’ “efforts to respect what they believe to be the cultural and personal desires of Somali women […] are actually based on misconceptions” (p. 530). The wish for vaginal tightness has to do with a fear among women that childbirth would leave them with “a gaping vaginal opening” (Johansen, 2018, p. 88), which should not be confused with a request for reinfibulation. This concern about “too wide” a vagina after childbirth is not uncommon among Western uninfibulated women who have delivered a baby (a situation which the plastic surgery market profits from; see, e.g., Barbara et al., 2017; Boddy, 2016). Consequently, there is reason to question figures on how many patients want reinfibulation after delivery. When researchers endeavor to establish how many women ask for an authentic reinfibulation, and not only vaginal tightness, studies show low figures. In Abdulcadir, McLaren, Boulvain, and Irion (2016), for example, only 8 out of 196 infibulated women requested reinfibulation (and all withdrew their request after counseling).

Traditionally, in East African countries, such as the Sudan and Somalia, infibulation is seen as the ultimate sign of virginity and chastity (Johansen, 2006, 2018, 2019; Johnsdotter, 2002). In addition, infibulation is associated with ideas that the narrow vaginal opening provides increased male sexual pleasure (Abdulcadir et al., 2016; Gruenbaum, 2001, 2006; Johansen, 2006, 2018; Johnsdotter, 2002; Magied, El Balah, & Kawther, 2000; Nour 2008). An interviewee in a study by Johansen (2018) said: “All men want tight women. We are so scared that if we are not tight enough, the man will find a new woman to marry, or take a younger lover” (p. 88; see also Johnsdotter, 2002).

This association between infibulation and male sexual pleasure is widespread among both men and women in Somalia and the Sudan. However, it is difficult to disentangle the idea from ideals of moral and religious purity, virginity and chastity, and even aesthetics. There might also be reason to distinguish between generalized ideas on the one hand and experiences among real men on the other. Penetrating an infibulated vagina might be painful for men on two levels: physically and psychologically. Penetrating too narrow a vaginal opening often results in wounds and infections in the penis (Almroth et al., 2001; Battle, Hennink, & Yount, 2017; Magied & Musa, 2004). Men might also suffer psychologically from inflicting pain during sexual intercourse. As a physician in the Sudan told anthropologist Gruenbaum (2006): “No sane man could enjoy sex that causes his wife pain” (p. 128). Somali men in a study in Sweden refuted the idea that infibulation would lead to increased sexual pleasure for them—the myth is upheld in women’s spheres, most of them said—and one of them became agitated when the issue was brought up:

This is so sick! It’s a problem everyone knows of. All men who plan to marry a woman who is pharaonically circumcised know what kind of hell they are to face. There are men who get into psychological problems because of this, as they are injured. There is hardly anywhere to get in, constantly he has to struggle to make the opening bigger. To talk about pleasure… it’s not even close to pleasure. It’s torture. It is nothing but a sheer hell. Many people who have moved to the cities and who are confronted with this problem, they choose to go to the hospital and ask for help to open. If it’s such a pleasure, why don’t they stick to the idea that the man should open the woman with his penis? (Johnsdotter, 2002, p. 150)

What might be the case here is a situation in which there is a generalized notion based on false beliefs at the community level, while men as individuals have opposite experiences, and singular women also have experiences that contradict the generalized idea (for examples, see Johnsdotter, 2002). Situations such as this have often been discussed in terms of “pluralistic ignorance”: when “many individuals express an opinion which is aligned with their subjective perception of group opinion rather than their actual beliefs” (Seeme, Green, & Kopp, 2019, p. 695; see also, e.g., Lambert, Kahn, & Apple, 2003).

Regarding infibulation and the widespread idea that an almost closed vagina is what men prefer for the sake of their sexual pleasure, three British medical researchers noted with surprise that “[m]any myths have been dispelled and no resistance has been met from the men who have, in fact, been very supportive of our policy on deinfibulation” (McCaffrey, Jankowska, & Gordon, 1995, p. 789). In a more recent study in Switzerland (Abdulcadir et al., 2016), the researchers had a similar experience: male partners among the women who wished to be reinfibulated supported the decision not to undergo the procedure. They concluded that “[o]ften, women had false beliefs regarding the fact that a man prefers, and experiences more pleasure with, a woman with closed genitalia” (p. 72). In their proposed counseling protocol, Abdulcadir et al. (2016) recommended that caregivers “[r]epeat explanations on possible false beliefs and myths” (p. 70), and they highlighted the role male partners can play in discussions about vulvar appearance and function after childbirth: “If possible, with the woman’s agreement, include the partner in the discussion, and encourage an exchange of views by the couple” (p. 70). These considerations could possibly be better developed in the model suggested by Brady et al. (2019), especially in their discussion about requests for reinfibulation. Potentially, caregivers have a role to play to dispel community-level myths and male partners could be presented as contributors to change, rather than relatives who hamper health-promoting decisions (see, e.g., Leval, Widmark, Tishelman, & Maina Ahlberg, 2004, for a critical discussion about midwives’ prejudices about Somali men in maternal care; see also Johnson-Agbakwu, Helm, Killawi, & Padela, 2014, for a discussion on Somali men’s role in obstetric decision-making processes in the U.S.).

## Migration and Life in the Diaspora

In the Target Article by Brady et al. (2019), they raise the issue of migration and life in the diaspora, but without integrating it into their model. They seem to want to avoid supporting either the view that FGC practices are upheld among migrant communities or the view that they are generally abandoned. They mention the possibility of cultural change regarding FGC after migration, but end with presenting a hypothesis: “experiences of discrimination and disadvantages in one’s new home could lead to the continued practice of FGC as a means of affirming one’s identity.” This is basically armchair speculation—while it might seem sound at an intuitive level, the support through empirical studies in Western host countries is weak.

By contrast, there is a growing body of research supporting the conclusion that major cultural change regarding FGC has taken place among the affected groups in Western host countries (e.g., Chu & Akinsulure-Smith, 2016; Cohen, Larsson, Hann, Creighton, & Hodes, 2018; Gele, Johansen, & Sundby, 2012; Gele, Kumar, Hjelde, & Sundby, 2012; Gele, Sagbakken, & Kumar, 2015; Johansen, 2019; Johnsdotter, 2002; Johnson-Agbakwu et al., 2014; Johnsdotter & Essén, 2015; Johnsdotter & Mestre i Mestre, 2015, 2017; Johnsdotter, Moussa, Carlbom, Aregai, & Essén, 2009; Koukoui, Hassan, & Guzder, 2017; Larsson, Cohen, Hann, Creighton, & Hodes, 2018; Shahawy, Amanuel, & Nour, 2019; Wade, 2016; Wahlberg, Johnsdotter, Selling, Källestål, & Essén, 2017a, 2017b).

Why is this important? It is relevant because as long as caregivers have expectations about upheld FGC activities, or imagine from the start of the conversation that their patient holds positive attitudes about FGC, this will have a negative impact on the relationship between caregiver and patient. If caregivers are convinced that the people they meet in clinical settings are secret proponents of FGC (as is often claimed in public discourse; see, e.g., Johnsdotter & Mestre i Mestre, 2017), this will affect their treatment of the persons with whom they communicate. Brady et al. (2019) touched upon the issue of mandatory reporting of FGC in some countries and concluded that such routines “may make some patients reluctant to discuss FGC with providers.” This is, in our view, underestimating the negative effects on communities of harsh anti-FGM policies. The situation in the UK is illustrative: It has perhaps the most advanced legal regulations toward professional groups when it comes to FGC, and among the measures is an absolute duty for healthcare providers to report any FGC found in a minor girl. These policies aim to identify illegal cases of FGC, but the “side effects” for the affected communities are severe (Creighton, Samuel, Otoo-Oyortey, & Hodes, 2019; Johnsdotter, 2019; Karlsen, Carver, Mogilnicka, & Pantazis, 2019; Lane, Johnson-Agbakwu, Warren, Budhathoki, & Cole, 2018). As reported by a research group in the UK after a study among Somalis in Bristol:

Women in our focus groups experienced FGM-safeguarding repeatedly in routine health care settings with midwives, GPs and health visitors. They believed medical staff prioritised extracting the information required for Government statistics over and above their health needs and without consideration of their trauma in connection with their past experiences of FGM. Participants said that health professionals repeatedly “put salt on the wound” caused by FGM through relentless and insensitive questioning, and “fixated” on FGM to the detriment of the patient in front of them. As a result, they reported avoiding medical care and/or approaching appointments with hostility and fear. (Karlsen et al., 2019, p. 7)

The model suggested by Brady et al. (2019) might work as an antidote to such situations in the clinic, as it might provide healthcare providers with a framework favoring nonjudgmental and sensitive attitudes when discussing FGC with their patients. But their model and discussion neglect the pervasiveness of such policies, as the effects of decades of harsh anti-FGM campaigning will be present in the minds of both caregiver and patient, and explicitly or implicitly influence the conversation. Their model and discussion regarding deinfibulation and its cultural context would benefit from engaging more directly with questions about cultural change in the diaspora and the negative effects of excessively harsh anti-FGM policies. If caregivers in Western host countries were better aware of migrants’ perspectives and the processes of attitude change among these communities, this could work as a starting point for building trust in the clinical encounter. As reported in an article published more than a decade ago about Somalis in the USA, women might feel that their immigrant communities do not receive credit for changes already made when it comes to reassessment of FGC practices: “women also felt their contribution to the elimination of the practice as African women had been overlooked” (Khaja, Barkdull, Augustine, & Cunningham, 2009, pp. 734–735). Lane et al. (2018), in an article about U.S. healthcare providers’ knowledge of FGC, underscored the importance of seeing this issue in the wider sociopolitical context, and claimed that “the need to improve providers’ knowledge about FGC is particularly acute at this time” (p. 962); this because fear and distrust among migrant communities that result from legislation and governmental surveillance “lead to poor health seeking behavior with devastating consequences” (ibid.).

## Conclusions

In conclusion, we are impressed by the nuanced, subtle, and truly sensitive model that was elaborated by Brady et al. (2019). If it is further developed, we would suggest that Brady et al. expand their discussion to include a more critical stance regarding possible medical complications of infibulation, the importance of taking age, length of stay in the host country, and life situation of the patient into consideration, and the opportunity to dispel community-level false beliefs in clinical conversations about FGC. Furthermore, their discussion would benefit from a more explicit migrant’s perspective—both regarding culturally changing of views on FGC among affected communities and the potential negative effects for clinical encounters that are influenced by harsh anti-FGM policies.
